# Celastrol alleviates diabetic vascular injury via Keap1/Nrf2-mediated anti-inflammation

**DOI:** 10.3389/fphar.2024.1360177

**Published:** 2024-05-31

**Authors:** Ning An, Rixiang Wang, Lin Li, Bingyu Wang, Huiting Wang, Ganyu Peng, Hua Zhou, Gen Chen

**Affiliations:** ^1^ The Affiliated Li Huili Hospital of Ningbo University, Health Science Center, Ningbo University, Ningbo, China; ^2^ Department of Pharmacology, Health Science Center, Ningbo University, Ningbo, China

**Keywords:** dFUS, Nrf2, inflammation, celastrol, angiogenesis

## Abstract

**Introduction:** Celastrol (Cel) is a widely used main component of Chinese herbal medicine with strong anti-inflammatory, antiviral and antitumor activities. In the present study, we aimed to elucidate the cellular molecular protective mechanism of Cel against diabetes-induced inflammation and endothelial dysfunction.

**Methods:** Type 2 diabetes (T2DM) was induced by db/db mice, and osmotic pumps containing Cel (100 μg/kg/day) were implanted intraperitoneally and were calibrated to release the drug for 28 days. In addition, human umbilical vein endothelial cells (HUVECs) were cultured in normal or high glucose and palmitic acid-containing (HG + PA) media in the presence or absence of Cel for 48 h.

**Results:** Cel significantly ameliorated the hyperglycemia-induced abnormalities in nuclear factor (erythroid-derived 2)-like protein 2 (Nrf2) pathway activity and alleviated HG + PA-induced oxidative damage. However, the protective effect of Cel was almost completely abolished in HUVECs transfected with short hairpin (sh)RNA targeting Nrf2, but not by nonsense shRNA. Furthermore, HG + PA reduced the phosphorylation of AMP-activated protein kinase (AMPK), the autophagic degradation of p62/Kelch-like ECH-associated protein 1 (Keap1), and the nuclear localization of Nrf2. However, these catabolic pathways were inhibited by Cel treatment in HUVECs. In addition, compound C (AMPK inhibitors) and *AAV9-sh-Nrf2* reduced Cel-induced Nrf2 activation and angiogenesis in db/db mice.

**Discussion:** Taking these findings together, the endothelial protective effect of Cel in the presence of HG + PA may be at least in part attributed to its effects to reduce reactive oxygen species (ROS) and inflammation through p62/Keap1-mediated Nrf2 activation.

## Introduction

Diabetic foot ulcers (DFUs) are caused by diabetes-induced damage to deep tissues, including the blood vessels and nerve endings of the lower extremities. Specifically, chronic DFU is characterized by a highly active inflammatory state that is the result of, in part, poor pathogen control and high concentrations of pro-inflammatory cytokines. Furthermore, high levels of ROS and poor angiogenesis are present in patients with DFUs (Bowling et al., 2015; Gallagher et al., 2024). Although wound care management is an established clinical field, the management of chronic diabetes-related skin lesions remains a major challenge.

Nuclear factor erythroid 2-related factor 2 (Nrf2) is a redox-sensitive transcription factor that regulates the transcription of many key antioxidant genes. Under conditions of oxidative stress, cysteine residues on Kelch-like ECH-associated protein 1 (Keap1) are oxidized by ROS, resulting in the release and activation of Nrf2, and its translocation to the nucleus (Dinkova-Kostova et al., 2002; Zhang and Hannink, 2003). In addition to the canonical pathway, p62 also interacts with the Nrf2-binding site of Keap1, competitively inhibiting the Keap1-Nrf2 interaction, which results in the expression of a series of genes encoding antioxidant proteins and anti-inflammatory enzymes (Komatsu et al., 2010; Lau et al., 2010). Nrf2 positively regulates *p62* gene expression, which suggests the existence of a positive feedback loop (Jain et al., 2010). Recently, an important role for Nrf2 in the prevention of DFUs has been reported (Xu et al., 2022). Nrf2-mediated antioxidant capacity appears to counteract the stress response that is induced by a highly active inflammatory state. Furthermore, the activation of Nrf2 protects against high glucose-induced apoptosis and cell damage in the kidney, heart, and blood vessels (Hashemi et al., 2023). Thus, the Nrf2 signaling pathway represents a therapeutic target for the cardiovascular complications of obesity and diabetes.

Substances derived from plants are an important source of new medicines. Thunder God Vine is an ancient herb that has been used in China for more than 2,000 years to treat chronic inflammatory diseases (Venkatesha and Moudgil, 2016). Celastrol (Cel, C_29_H_38_O_4_), a pentacyclic triterpene, was originally extracted from *Trypterygium wilfordii Hook F*. and shows potential as a treatment for chronic diseases, such as Parkinson’s disease, Alzheimer’s disease, atherosclerosis, osteoarthritis, and rheumatoid arthritis (Allison et al., 2001; Pang et al., 2010; Gu et al., 2013; Kim et al., 2013; Choi et al., 2014). In addition, a recent study of Cel demonstrated that it ameliorates some metabolic diseases, such as obesity (Feng et al., 2019), insulin resistance (Ma et al., 2015), and suppresses cardiac and renal fibrosis (Guo et al., 2017; Ye et al., 2020). Cel has powerful antioxidant (Yuan et al., 2023) and anti-inflammatory effects (Hu et al., 2017; An et al., 2020; Shirai et al., 2023) that are mediated through upregulation of the Nrf2/antioxidant enzyme pathway (Nakayama et al., 2020; Qing et al., 2023). However, the effects of Cel on DFUs and the associated changes in angiogenesis have not been investigated. Therefore, in the present study, we aimed to determine whether Cel ameliorates hyperglycemia-induced endothelial dysfunction through the activation of Nrf2-related exogenous antioxidants, and to characterize its effects on the related signaling pathways.

## Materials and methods

### Animal procedures

Diabetic db/db mice and their control littermates, db/dm, were obtained from the GemPharmatech Co., Ltd. Before the commencement of the study, the mice were acclimated to their new environment for 4–6 days. They were housed at a temperature of 21°C ± 2°C and a relative humidity of 50% ± 15%, under a 12 h light-dark cycle. The fasting blood glucose concentrations of the mice were measured at the beginning of the study (Supplemental Table S1). AAV9 harbouring Nrf2 shRNA (*AAV9-CDH5-sh-Nrf2*) and control vector (*AAV9-CDH5-Scrambled*) were injected intravenously into tail veins of 7 weeks old male db/db or db/dm mice respectively. To ensure the effect of 1 × 10^12^ vg of virus infection, we did the subsequent experiment 2 weeks later. ALZET^®^ Osmotic Pumps (Model 2004) containing Cel (100 μg/kg/day, C0869, Sigma-Aldrich) (Fang et al., 2019) was implanted intraperitoneally, and then were calibrated to release the drug for 28 days (Figure 4E). The body mass and food intake of the mice were recorded every 3 days during the treatment period. All the mice were fasted for 12 h and subjected to glucose tolerance testing and insulin tolerance testing. For the analysis of signaling, bafilomycin A1 (10 mg/kg/2d, i. p., S1413, Selleck) and compound C (10 mg/kg/2d, i. p., S7306, Selleck) were administered. The procedures used in the study complied with the animal ethics guidelines of the institution and were approved by the Institutional Animal Care and Use Committee of Ningbo University, China.

### Cell culture

Human umbilical vein endothelial cells (HUVECs), widely used for the study of vascular function and repair (Guixé-Muntet et al., 2017; Cherubini et al., 2023), were purchased from Lonza (Basel, Switzerland) and cultured at 37°C in a 5% CO_2_-containing humidified incubator using endothelial cell growth medium-2 (EGM-2, CC-3156, and CC-4176, Lonza) containing normal glucose (NG, 5.5 mM) or HG + PA (33 mM HG + 200 μM PA) (Alnahdi et al., 2019; Huang et al., 2021), with or without 100 nM Cel, for 48 h (Li et al., 2017; Ma et al., 2020). Fifth-to-seventh-generation subconfluent cells were used for the experiments. Mannitol (MAN, 33 mM: 5.5 mM of glucose +27.5 mM of D-mannitol; M4125, Sigma-Aldrich) was used as an osmolarity control for HG. For the analysis of the signaling pathways, MG132 (5 μM for 3 h), bafilomycin A1 (Baf A1, 10 nM for 12 h), or compound C (10 μM for 12 h) were added.

### Plasmids

HUVECs were transfected overnight with Nrf2-targeting shRNA (sc-37030, Santa Cruz) or nonsense shRNA at an MOI of 100× PFU/cell, and then the culture medium was replaced after 24 h. After 48 h, the expression of Nrf2 was measured by Western blot analysis.

### Immunoblotting analysis

Briefly, samples containing 30 μg protein were separated by SDS-PAGE on Tris-glycine gels and then transferred to polyvinylidene difluoride membranes. The membranes were blocked and incubated the primary antibody or secondary antibody [HRP-goat-anti-mouse (115-035-003, Jackson) or HRP-goat-anti-rabbit (111-035-003, Jackson)]. Immunoreactive bands were visualized using Pierce ECL Western blotting substrate (WBKLS0500, Millipore).

The HUVECs were lysed and obtained cytoplasmic and nuclear lysates using the Keygen Nuclear-Cytosol Protein Extraction Kit from Nanjing KeyGen Biotech. Co., Ltd.

The primary antibodies used to probe the membranes were against p62 (sc-48402, 1:1,000), Lc3 (sc-271625, 1:1,000) (Santa Cruz Biotechnology), p-AMPK (2531S, 1:1,000), AMPK (2532S, 1:1,000) (Cell Signaling Technology), Nrf2 (66504-1, 1:1,000), Keap1 (10503-2, 1:1,000), β-actin (20536-1, 1:1,000), GAPDH (60004-1, 1:5,000), and Lamin b (66095-1, 1:1,000) (Proteintech). ImageQuant 5.2 software (Molecular Dynamics) was used to quantitatively analyze the expression of specific proteins, and β-actin was used as the loading control.

### Immunoprecipitation

IGEPAL CA-630 buffer [150 mM NaCl, 50 mM Tris-HCl, 2 mM EDTA, 1 μM leukopeptin (L5793, Sigma-Aldrich), 50 mM NaF, and 0.1 μM aprotinin (SRE0050, Sigma-Aldrich), 1% IGEPAL CA-630 (I8896, Sigma-Aldrich), pH 7.4] was used to lyse the HUVECs. After co-immunoprecipitation, the precipitates were washed five times with TBS. They were then eluted with glycine-HCl (0.1 M, pH 3.5) and the immunoprecipitates were subjected to immunoblotting using specific primary antibodies.

### RNA isolation and quantitative real-time-PCR (qRT-PCR)

RNA was extracted from HUVECs using TRIzol reagent (9,108, Takara Bio Inc.), according to the manufacturer’s instructions. Next, total RNA (2 µg) was reverse transcribed into cDNA by using GoScript Reverse Transcription Kit (Promega, A5001). Quantitative RT-PCR analysis was performed using PowerUp SYBR Green Master Mix (Thermo Fisher Scientific, A25918). The relative expression of each gene was quantified using the 2^−ΔΔCT^ method and normalized to the expression of *Actb*. The specific primer sequences used for qRT-PCR are listed in Supplementary Table S2.

### Cell counting kit (CCK)-8 assay

HUVECs (1.0 × 10^4^ per well) were cultured in 96-well plates for 24 h and treated with 25, 50, 75, 100, 200, 500, 750, or 1,000 nM Cel for 24 or 48 h, and their viability was assessed using the CCK-8 method. The absorbance of each well was measured using a microplate reader at 450 nm.

### Quantitative determination of oxidative stress (dihydroethidium assay)

HUVECs treated with HG + PA were stained with dihydroethidium (DHE, D7008, Sigma-Aldrich) probes to measure their ROS concentrations. DHE is cell permeable and able to react with superoxide to form ethidium, which in turn intercalates with DNA and produces nuclear fluorescence. HUVECs were seeded on 24-well plates and treated with HG in presence or absence Baicalin for 72 h and then incubated with 5 μM DHE in DMSO for 30 min at 37°C. Nuclear DHE positive staining indicates superoxide generation in cells. The fluorescence intensity was observed with a computer-assisted microscope (EVOS, Thermo Fisher Scientific).

### TUNEL staining

HUVECs were stained using an *In situ* Cell Death Detection kit (11684795910, Roche), according to the manufacturer’s protocol. Cultured HUVECs were fixed (4% paraformaldehyde for 30 min) and permeabilized (0.1% Triton-X 100 for 10 min) in 6-well plates. After washing with PBS, cells were incubated with 50 µL tunel reaction mixture for 60 min at 37°C. After washing, the nuclei were stained with DAPI. The stained cells were then examined using a confocal laser scanning microscope (TCS SP8, Leica, Wetzlar, Germany).

### *In vitro* angiogenesis (tube formation) assay

The angiogenic activity of the HUVECs was assessed using a Matrigel tube formation assay. Briefly, HUVECs were scattered on the surface of Matrigel (Corning, 354234) and incubated in a cell incubator at 37°C for 24 h, followed by staining with the cell permeability dye Calcein AM (Corning, 354216) for 30 min. The formation of capillary-like tubes was identified using a computer-assisted microscope (EVOS, Thermo Fisher Scientific), the lengths of the tubes were calculated using ImageJ software (National Institutes of Health, Bethesda, MD, United States), and the mean values for the replicate wells were calculated.

### Aortic ring assays

To establish a direct action of Cel on vascular, thoracic aortae from db/db and db/dm mice infected with *AAV9-CDH5-sh-Nrf2* or *AAV9-CDH5-Scrambled* after 4-week treatment. Mice were surgically isolated, cleaned, and sectioned to form 0.5 mm rings. The lentivirus-mediated gene transfer (*Lv-CDH5-sh-Nrf2*) and vector (*Lv-sh-Scrambled*) were also respectively transfected with aortic rings from db/db and db/dm mice infected with *AAV9-CDH5-sh-Nrf2* or *AAV9-CDH5-Scrambled*. Next the rings were embedded and cultured in 96-well plates containing type I collagen (08-115, Millipore, 1 mg/mL) as previously described (Aplin et al., 2008; Baker et al., 2011). Then the medium was removed and replaced with either mannitol (33 mM: 5.5 mM of glucose +27.5 mM of D-mannitol) or HG + PA (33 mM HG + 200 μM PA) in the presence or absence of Cel (100 nM). Pre-treatment with Cel and VEGF were performed every day to analyze the role of the key signaling pathway. The number of endothelial microvessels that grew out of the rings was counted during the exponential growth phase to assess angiogenesis. Before the regression phase, the rings were fixed and immunofluorescence-stained for CD31 (ab281583, Abcam). Photomicrographs were obtained after 12 days, and the total number of branches was counted using ImageJ.

### Scratch assay of wound healing

Cell migration was assessed using a scratch assay, as previously described (Das et al., 2018). Cells were seeded into six-well plates and cultured overnight until a confluent monolayer formed, which was then scratched using a 200 µL pipette tip. Before and 36 h after this injury, images of the damaged cell monolayers were captured using a microscope (EVOS, Thermo Fisher Scientific) and quantified using ImageJ software. All the experiments were performed in the presence of mitomycin-C (Jain et al., 2019) (10 μM, Selleck Chemicals, S8146), which inhibits cell proliferation.

### Wound healing assay in mice

General anesthesia was induced in mice by the inhalation of 2% isoflurane. Full-thickness wounds were created to the shaved dorsal skin of db/db and db/dm mice infected with *AAV9-CDH5-sh-Nrf2* or *AAV9-CDH5-Scrambled* using 8 mm skin biopsy punches. Skin wound edge injection of the *Lv-CDH5-sh-Nrf2* and control vector *Lv-Scramble*, and then each of the wounds were treated with DMSO, Cel (100 nM) with a diameter of 10 mm, and bandaged with sterile gauze. The subsequent wound closure was assessed daily (Li et al., 2015). The formula for calculating the remaining open wound area was as follows: wound area remaining open (%) = (open area on the indicated day/original wound area) × 100%. Five-micrometer-thick paraffin-embedded tissue sections were prepared, dewaxed, rehydrated, and stained with immunofluorescence of keratin 14 (ab119695, Abcam) and CD31 (ab281583, Abcam) respectively. After washing, the sections were incubated with secondary antibodies [AlexaFluor 647-conjugated anti-rabbit (ab150079, Abcam) in different tissue sections. The re-epithelialization ratio (leading edge ratio) was calculated as [(a + b)/c] × 100% (shown in Figure 4F; a and b are the lengths of the leading edges, and c is the initial length of the wound) (Safferling et al., 2013).

### Immunofluorescence

Immunofluorescence was used to identify areas of CD31, VCAM-1, and p65 expression in the aortic wall. Eight-micrometer-thick paraffin sections were prepared and incubated with anti-CD31 antibody (ab281583, Abcam), VCAM-1 antibody (ab134047, Abcam), or p65 antibody (66535-1, Proteintech). After washing, the sections were incubated with secondary antibodies [AlexaFluor 647-conjugated anti-rabbit (ab150079, Abcam) or AlexaFluor 488-conjugated anti-mouse (ab150113, Abcam)] at room temperature for 60 min. DAPI was used to label the nuclei. The CD31 or VCAM-1-stained area were examined using a confocal laser scanning microscope (TCS SP8, Leica, Wetzlar, Germany) and measured using ImageJ software. Data are presented as the percentages of the total area that was immunostained.

To quantitatively analyze the nuclear localization of Nrf2 in HUVECs. Cultured HUVECs were fixed (4% paraformaldehyde for 30 min), permeabilized (0.5% Triton-X 100 for 30 min) and blocked (5% BSA for 2 h) in 6-well plates. Then, cells were incubated with rabbit anti-Nrf2 primary antibody (1:200, 66504-1, Proteintech) overnight at 4°C. After washing, the cells were then incubated with secondary antibody (AlexaFluor 488-conjugated anti-mouse, ab150113, Abcam). The nuclei were stained with DAPI. The results were photographed via a confocal laser scanning microscope (TCS SP8, Leica, Wetzlar, Germany). The colocalization of Nrf2 and DAPI was assessed in randomly selected cells.

### Statistical analysis

All the analyses were performed with the investigator being blinded to the groups of mice or cultured cell treatments. Statistical comparisons were made using the two-tailed Student’s *t*-test for two experimental groups or the one-way analysis of variance (ANOVA) for multiple groups. Statistical analyses were performed using GraphPad Prism.

## Results

### Celastrol attenuates HG + PA-induced inflammation and apoptosis in HUVECs

**FIGURE 1:**
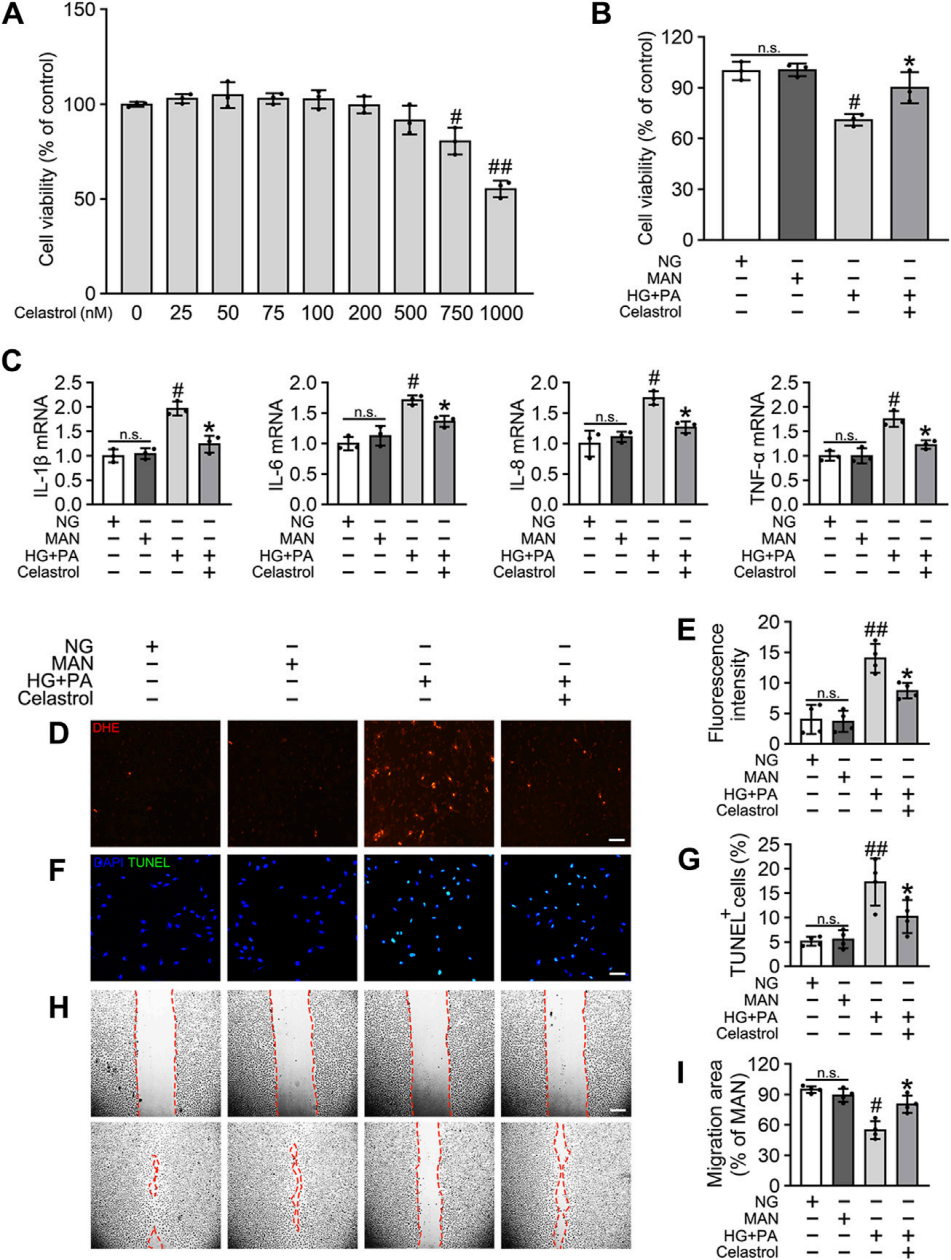
Cel attenuates HG + PA-induced inflammation and apoptosis in HUVECs. **(A)** The proliferation of HUVECs was monitored using a CCK-8 assay after exposure to different cultured conditions for 48 h. Values displayed are means ± SD of independent experiments. *p < 0.05 or **p < 0.01 vs. absence of Cel. **(B–I)** HUVECs cultured either in NG or HG + PA medium in the presence or absence of Cel for 48 h, MAN served as the osmotic control for the HG. **(B)** Shows cell viability of different groups. **(C)** The mRNA expression and quantitation of NF-κB downstream target genes were evaluated by sqRT-PCR. **(D)** Superoxide was determined with the fluorescent indicator DHE, Scale bars = 120 μm. **(E)** The quantitative analysis of fluorescent intensity in at least 4 separate fields. **(F)** TUNEL assay show that the apoptotic cells were labeled with green, and nuclei were stained with DAPI (blue). Scale bars = 100 μm. **(G)** The quantitative analysis of TUNEL^+^ cells in at least 4 separate fields. **(H)** A scratch wound healing assay was performed in the presence of Mitomycin-C (10 μM). Cell monolayers were imaged at 0 and 36 h after wounding. Red vertical lines indicate the wound area borders. Scale bar = 65 µm. **(I)** Cell migration distances were measured based on the data. Data shown in graphs **(B–I)** represent the means ± SD of independent experiments. n. s = not significant, #*p* < 0.05 vs. HUVECs expose to MAN; *p < 0.05 vs. HUVECs expose to HG + PA.

To determine the effects of Cel on the HG + PA-induced defects in HUVECs, we first evaluated the cytotoxicity of Cel *in vitro*. We found that concentrations of Cel <400 nmol/L caused no obvious cytotoxicity, whereas there was significantly lower cell viability when the concentration of Cel exceeded 750 nmol/L (Figure 1A). This is consistent with the effects of Cel to impair vascular growth in tumors at high concentrations (Pang et al., 2010). Furthermore, we found that the deleterious effect of HG + PA on the viability of HUVECs was significantly ameliorated by 100 nM Cel (Figure 1B). It has been reported that HG + PA-induced endothelial dysfunction is mediated through multiple mechanisms, including oxidative stress and pro-inflammatory responses (Xie et al., 2008). We demonstrated that HG + PA-induced inflammatory activity, reflected in the upregulation of the pro-inflammatory cytokines IL-1β, IL-6, IL-8, and TNF-α, was inhibited by Cel treatment (Figure 1C). In addition, we assessed the effect of Cel on oxidative stress in the endothelial cells using DHE. HG + PA treatment of HUVECs resulted in a significant increase in superoxide production, and this was attenuated by co-treatment with Cel (Figures 1D,E). HG + PA also induced a high level of apoptosis in HUVECs, as indicated by a larger proportion of TUNEL-positive cells. However, this HG + PA-induced apoptosis was significantly attenuated by Cel (Figures 1F,G). Furthermore, the migration of HG + PA-exposed HUVECs was significantly impaired, but this defect was significantly ameliorated by Cel (Figures 1H,I).

### Celastrol increases the activity of Nrf2 in HG + PA-treated HUVECs

**FIGURE 2:**
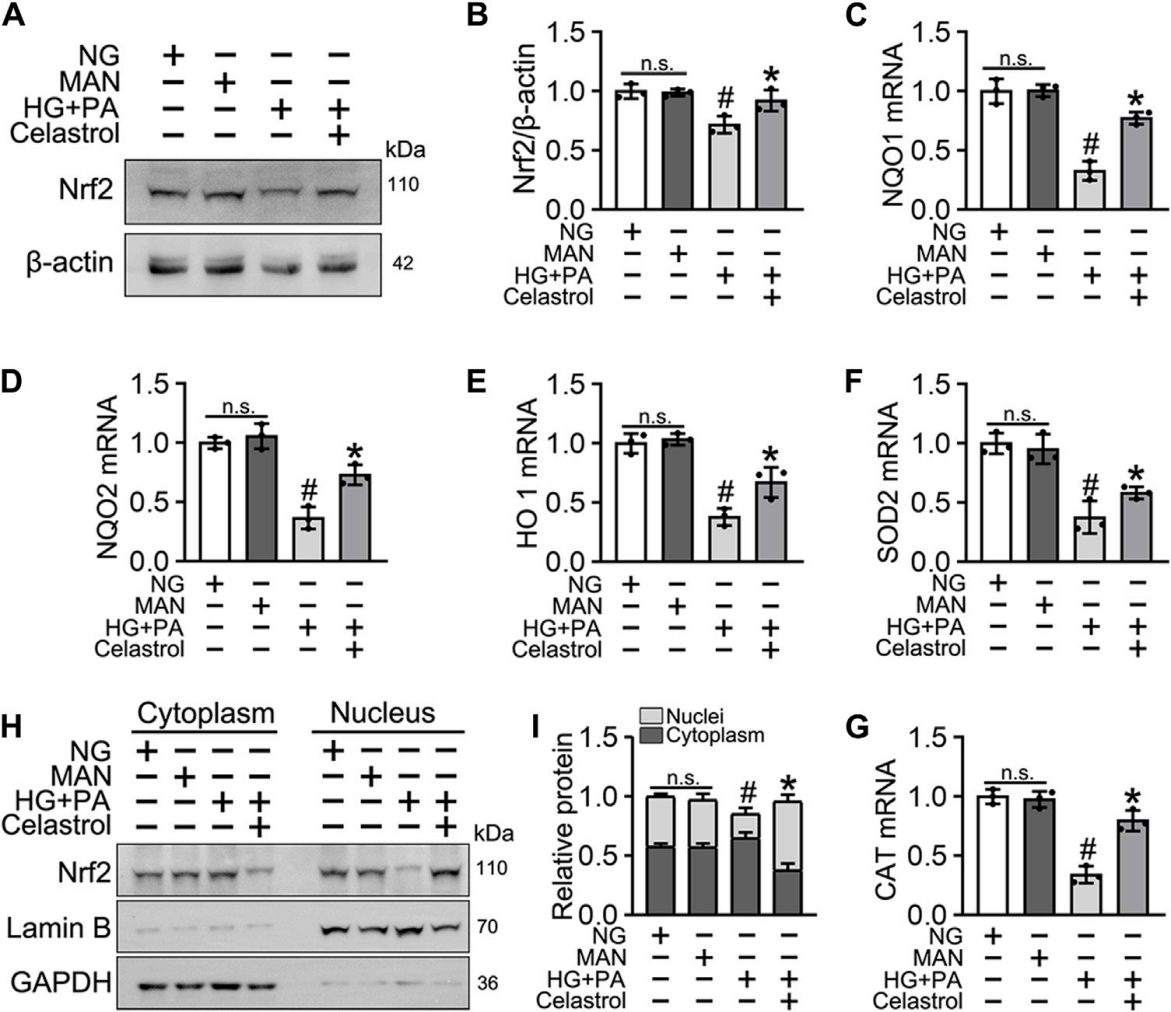
Cel enhanced the activity of Nrf2 in HG + PA-triggered HUVECs. HUVECs cultured either in NG or HG + PA medium in the presence or absence of Cel for 48 h, MAN served as the osmotic control for the HG. **(A)** The Nrf2 was evaluated by Western blot. **(B)** The quantitative analysis of immunoblots. **(C–G)** The mRNA expression and quantitation of Nrf2 downstream target genes were evaluated by sqRT-PCR. **(H)** Nuclear Nrf2 protein expression was measured using Western blotting. **(I)** The quantitative analysis of immunoblots. Data shown in the graphs represent the means ± SD of independent experiments. n. s = not significant, #*p* < 0.05 vs. HUVECs expose to MAN; *p < 0.05 vs. HUVECs expose to HG + PA.

Nrf2 plays a key role in the cellular response to oxidative stress (Jyrkkänen et al., 2008). Therefore, we speculated that Cel may ameliorate HG + PA-induced endothelial damage by activating Nrf2 and its downstream target genes. Nrf2 protein expression was downregulated by the HG + PA treatment, but consistent with previous findings (Li et al., 2017), the Nrf2 protein expression was significantly restored by Cel treatment (Figures 2A,B). Importantly, we found that the mRNA expression of the Nrf2 target genes *NQO1*, *NQO2*, *HO1*, *SOD2*, and *CAT* was significantly reduced by HG + PA treatment, but this was corrected by Cel treatment (Figures 2C–G). In addition, the nuclear localization of Nrf2 was significantly reduced by HG + PA treatment, but this did not occur in the presence of Cel (Figures 2H,I). The nuclear localization of Nrf2 in HUVECs was further characterized using immunofluorescence (Supplementary Figure S1A), which showed similar results. Overall, these results demonstrate that Cel restores Nrf2 protein expression and promotes the nuclear enrichment of Nrf2. Furthermore, consistent changes in Nrf2 target gene expression accompany the effects of Cel on Nrf2 nuclear localization, indicating that Cel activates the transcriptional function of Nrf2, ameliorates HG + PA-induced oxidative stress, and reduces the inflammatory response.

#### Celastrol ameliorates HG + PA-induced oxidative stress and inflammation through the activation of Nrf2 in HUVECs

**FIGURE 3:**
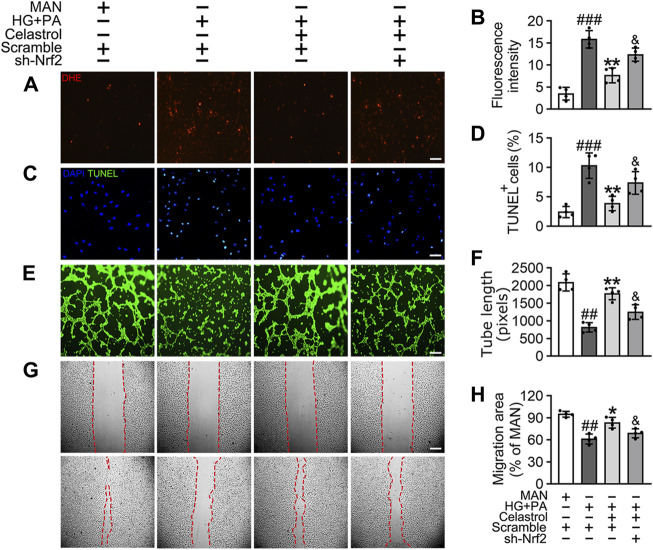
The protective effect of Cel on endothelial cells was mediated through Nrf2. HUVECs were transfected with or without Nrf2 shRNA (sh-Nrf2) or scramble shRNA (scramble) and then exposed to HG + PA medium in the presence or absence of Cel for 48 h. **(A)** Superoxide product test assay of HUVECs treated with the fluorescent indicator DHE, Scale bars = 120 μm. **(B)** The quantitative analysis of fluorescent intensity in at least 4 separate fields. **(C)** TUNEL assay: the apoptotic cells were labeled with green, and nuclei were stained with DAPI (blue). Scale bars = 100 μm. **(D)** The quantitative analysis of TUNEL^+^ cells in at least 4 separate fields. **(E)** Capillary-like tube formation was assessed by matrigel angiogenesis assay in HUVECs. Scale bars = 85 μm. **(F)** Quantification of the tube length, and images of tube morphology were taken in four random microscopic fields per sample. **(G)** A scratch wound healing assay was performed in the presence of Mitomycin-C (10 μM). Cell monolayers were imaged at 0 and 36 h after wounding. Red vertical lines indicate the wound area borders. Scale bar = 65 µm. **(H)** Cell migration distances were measured based on the data. Data shown in the graphs represent the means ± SD of independent experiments. ###*p* < 0.001 vs. HUVECs expose to MAN; **p < 0.01 vs. HUVECs expose to HG + PA, and *p* < 0.05 vs. HUVECs expose to HG + PA treatment with Cel.

To confirm the role of Nrf2 in the endothelial protective effects of Cel, we knocked down the expression of Nrf2 using specific shRNA. We found that even in the presence of Cel, Nrf2 shRNA treatment reduced the expression of a series of antioxidant-related genes (*NQO1*, *NQO2*, *HO1*, *CAT*, and *SOD2*) and increased the production of pro-inflammatory cytokines (IL-1β, IL-6, IL-8, and TNF-α) (Supplementary Figure S2). Notably, Cel significantly reduced HG + PA-induced superoxide generation (Figures 3A,B) and apoptosis (Figures 3C,D) in HUVECs, but this was impaired by Nrf2 shRNA co-treatment. In addition, the impairments in tube formation (Figures 3E,F) and migration (Figures 3G,H) of the HUVECs were also significantly ameliorated by Cel, but this effect was abrogated by Nrf2 shRNA co-administration. However, we found that Cel had a very small effect to enhance tube formation by the HUVECs under basal conditions (Supplementary Figure S3A). These results suggest that the Cel-induced activation of Nrf2 promotes cell proliferation and migration, and inhibits the apoptosis induced by HG + PA treatment.

#### The Celastrol-induced activation of Nrf2 improves angiogenic function in the presence of T2DM-associated endothelial dysfunction

**FIGURE 4:**
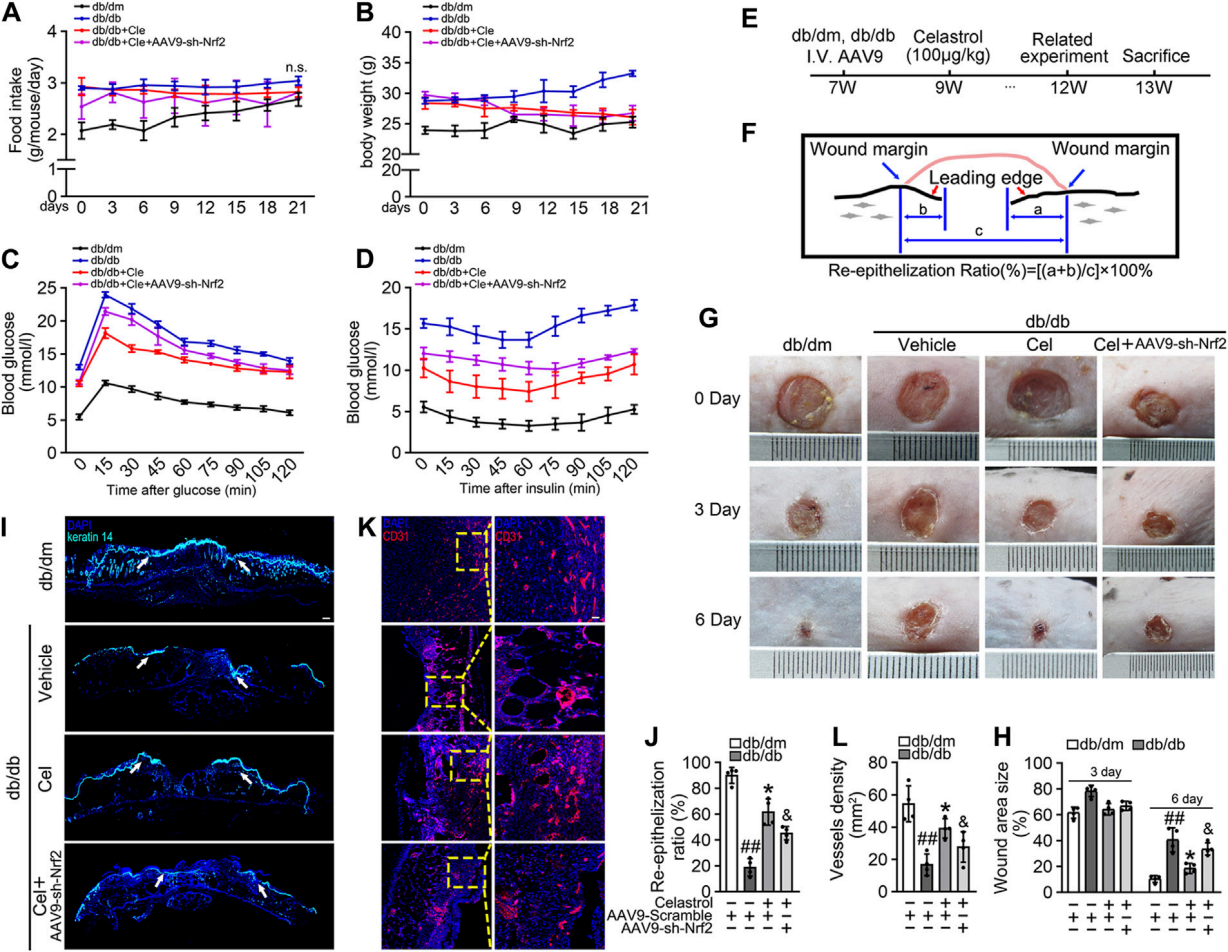
Celastrol-mediated Nrf2 ameliorates wound healing in db/db mice. **(A)** During the treatment period, the food intake of the mice was measured every 3 days **(B)** During the treatment period, the body weight of mice was measured every 3 days **(C)** Glucose tolerance test (GTT). After 21 days of administration of celastrol, the fasting hyperglycemia of the mice was reduced, and the glucose tolerance was also improved, which was related to the reduction of plasma glucose level in the fasting state and the response to glucose load. **(D)** Insulin tolerance tests (ITT). After 21 days of celastrol administration, mice showed improved insulin resistance and lowered blood glucose levels in response to glycemic load. **(E)** Design of the study. **(F)** Schematic diagram about **(I)**, the lengths of leading edges and the initial wound length were defined as indicated. **(G)** Images of skin wounds from db/dm mice and db/db mice (*n* = 4). **(H)** The wound area size (%) was measured for 6 days **(G)**. **(I)** The skin sections were harvested and followed by immunofluorescence staining of keratin 14 (cyan). Scale bar = 1 mm. **(J)** Measurement of reepithelization ratio (leading edge ratio) in wound area **(I)**. **(K)** The skin sections were harvested and followed by immunofluorescence staining of CD31 (red). Scale bar = 25 μm. (L) Quantification of the CD31^+^ capillary density in wound area **(K)**. Data shown in the graphs represent the means ± SD of independent experiments. ##*p* < 0.01 vs. db/dm mice; *p < 0.05 vs. db/db mice, and *p* < 0.05 vs. db/db mice treatment with Cel.

We next aimed to evaluate the effects of Cel and Nrf2 activity on the angiogenic function of endothelial cells in db/db mice (Figure 4E). Cel reduced the food intake and body mass of the diabetic mice (Figures 4A,B). It also rapidly reduced the circulating glucose concentrations (Figure 4C) and improved the insulin sensitivity (Figure 4D) of fasted diabetic mice, as demonstrated by glucose and insulin tolerance testing. However, transfected with *AAV9-sh-Nrf2* did not significantly impair the effects of Cel on body mass or blood glucose concentration (Figures 4B,D). To further assess the role of Nrf2 activity in the endothelial protection induced by Cel, we used an *in vivo* model of skin wound healing in mice, in which chronic inflammation inhibits endothelial function and subsequent wound healing (Safferling et al., 2013; Li et al., 2015). We found that *AAV9-sh-Nrf2* delayed the closure of wounds (Figures 4G,H) and was significantly less re-epithelialization in db/db mice than in the normal db/dm mice (Figures 4I,J). Furthermore, as model of skin wound healing in mice, we found that CD31^+^ capillary density was lower in regenerated skin tissue from db/db mice than in skin from db/dm mice, and this was especially evident in db/db mice transfected with *AAV9-sh-Nrf2* (Figures 4K,L).

**FIGURE 5:**
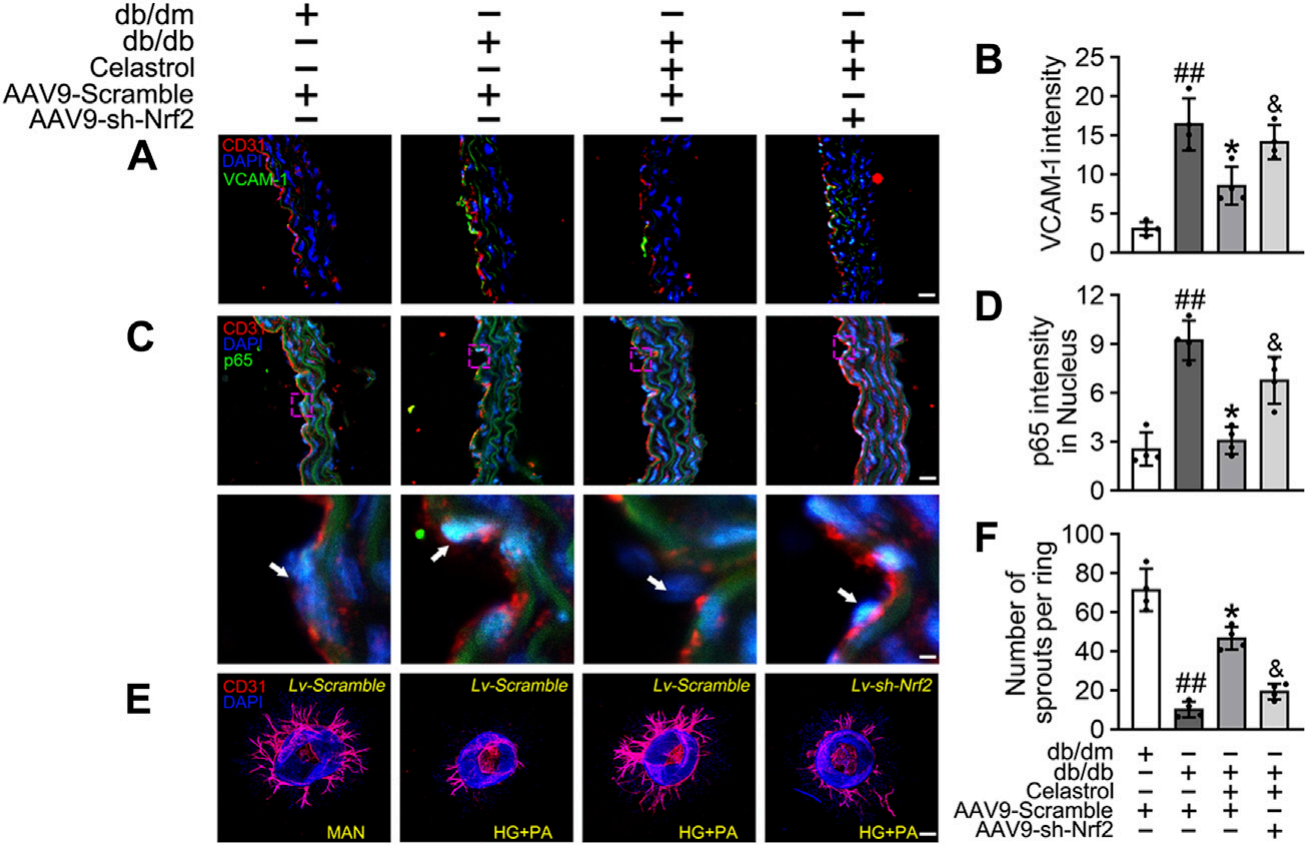
Cel attenuated inflammation and promoted angiogenesis in db/db mice. AAV9 harbouring Nrf2 shRNA (*AAV9-CDH5-sh-Nrf2*) and control vector (*AAV9-CDH5-Scrambled*) were injected intravenously into tail veins of 7 weeks old male db/db or db/dm mice respectively. Osmotic pumps containing Cel (100 μg/kg/day) was implanted intraperitoneally and were calibrated to release the drug for 28 days in db/db mice. **(A)** Representative confocal images of inflammation stress marker VCAM-1 in aortal vascular endothelium. The red area (CD31) represented endothelium, the green area represented VCAM-1 positive staining and the nucleus was blue. Scale bars = 40 μm. **(B)** Quantification of the number of VCAM-1 staining. **(C)** The presence of immunofluorescence with CD31 and p65 of aortal vascular endothelium. Scale bars = 40 μm. **(D)** The quantitative analysis of p65^+^ in the nucleus of aortal vascular endothelium. **(E)** Representative images of aortic rings were transfected with *Lv-CDH5-sh-Nrf2* or *Lv-Scramble* and then exposed to MAN and HG + PA in the presence or absence of Cel (100 nM). All aortic rings cultured with VEGF (30 ng/mL). Scale bars = 350 μm. **(F)** Quantification of the number of sprouts. Data shown in the graphs represent the means ± SD of independent experiments. ##*p* < 0.01 vs. db/dm mice; *p < 0.05 vs. db/db mice, and *p* < 0.05 vs. db/db mice treatment with Cel.

Subsequently, using vascular cell adhesion molecule (VCAM-1) as a marker of inflammatory stress, we showed that Cel treatment attenuated HG + PA-induced inflammation in HUVECs, whereas transfected with *AAV9-sh-Nrf2* worsened this (Figures 5A,B). In parallel, immunofluorescence analysis showed that the p65 expression in the aortic endothelial cell nuclei of diabetic mice was higher than that of normal mice. This effect was significantly ameliorated by Cel treatment, but the effect of Cel was abrogated by *AAV9-sh-Nrf2* administration (Figures 5C,D). The *ex vivo* aortic ring sprouting assay was used to further assess the protective effects of Cel on the endothelium. Aortic rings from male db/db or db/dm mice were firstly transfected with *Lv-sh-Nrf2* or *Lv-Scramble*, then cultured in HG + PA or MAN-containing medium in the presence or absence of Cel. In MAN medium, a well-structured microvessel network with clearly defined tubules and regular branching developed. By contrast, aortic rings cultured in HG + PA medium showed a significant impairment in budding, which was ameliorated by Cel, but this effect of Cel was abolished by *Lv-sh-Nrf2* (Figures 5E,F). Consistent with the *in vitro* findings, there was no significant improvement after Cel treatment in aortic ring sprout density under basal conditions (Supplementary Figure S3B). Taken together, these results indicate that the Cel-induced increase in Nrf2 activity inhibited oxidative stress and NFκB activity, thereby significantly ameliorating T2DM-associated endothelial dysfunction.

### Celastrol activates Nrf2 via the p62-Keap1 pathway, both *in vitro* and *in vivo*

Cel is thought to improve skeletal muscle and bone function through upregulation of the AMPK signaling pathway (Abu et al., 2020; Li et al., 2020), and it also ameliorates angiotensin II-mediated vascular smooth muscle cell senescence (Xu et al., 2017) and osteoarthritis (Feng et al., 2020) through the induction of autophagy. In addition, the function of Nrf2 has been reported to be regulated by the p62/Keap1/Nrf2 signaling pathway, the activation of which liberates Nrf2 from Keap1 and permits it to translocate to the nucleus (Zhang et al., 2021).

**FIGURE 6:**
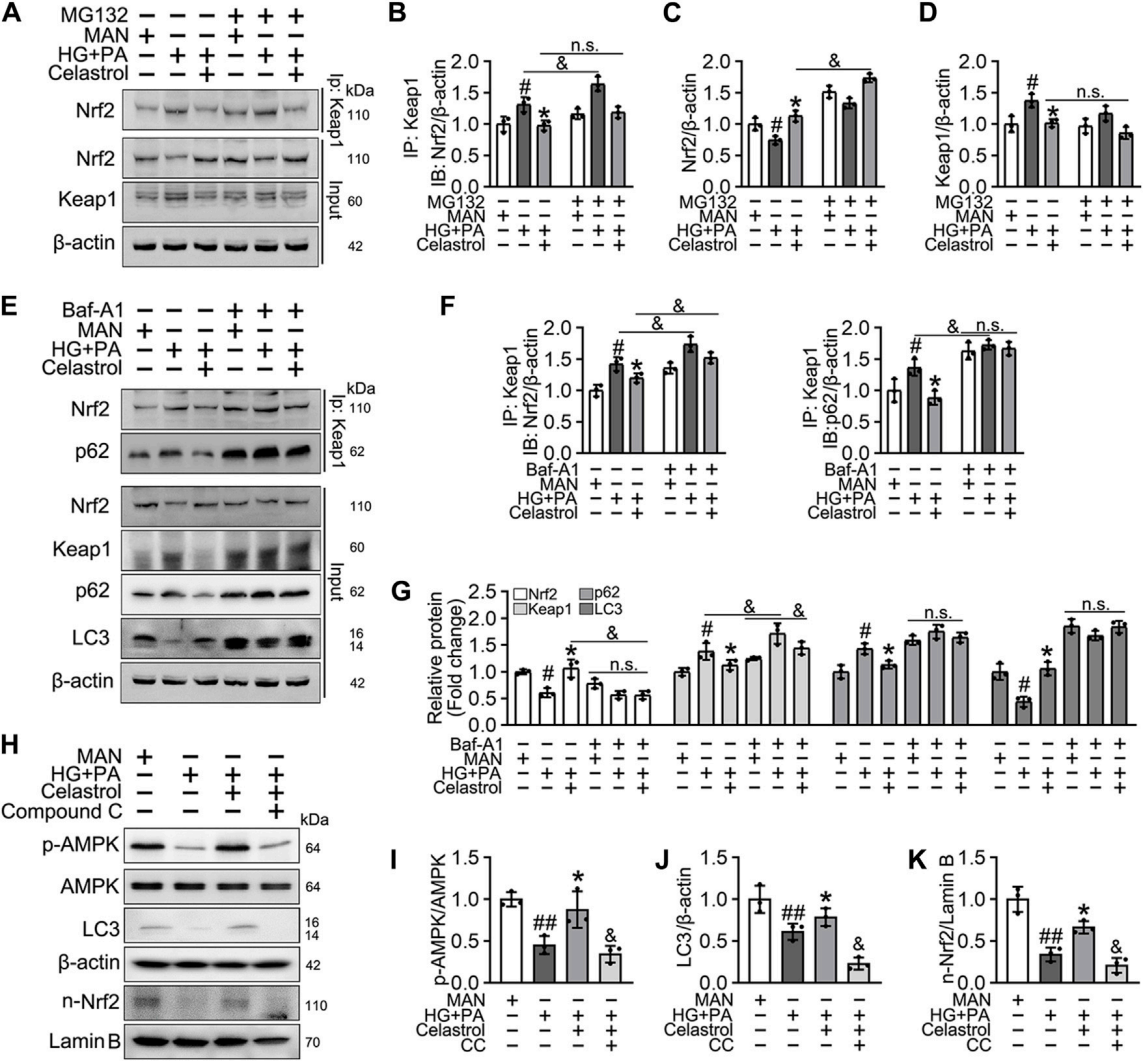
Cel activated Nrf2 via AMPK/p62/keap1 pathway *in vitro.* **(A–D)** HUVECs were cultured either in MAN (5.5 mM) and HG + PA (33 mM HG+200 μM PA) medium in the presence or absence of Cel (100 nM) or MG132 (5 μM) for 48 h. The cell lysates were subjected to immunoprecipitation with the Keap1 antibody, followed by immunoblotting with the indicated antibodies. The quantitative analysis of immunoprecipitation **(B)** and immunoblots **(C and D)**. Data shown in the graphs represent the means ± SD of independent experiments. #*p* < 0.05 vs. HUVECs expose to MAN; *p < 0.01 vs. HUVECs expose to HG + PA, and *p* < 0.05, n. s = not significant. **(E–G)** HUVECs were cultured either in MAN (5.5 mM) and HG + PA (33 mM HG+200 μM PA) medium in the presence or absence of Cel (100 nM) or Baf-A1 (20 nM) for 48 h. The cell lysates were subjected to immunoprecipitation with the Keap1 antibody, followed by immunoblotting with the indicated antibodies. The quantitative analysis of immunoprecipitation **(F)** and immunoblots **(G)**. Data shown in the graphs represent the means ± SD of independent experiments. #*p* < 0.05 vs. HUVECs expose to MAN; *p < 0.01 vs. HUVECs expose to HG + PA, and *p* < 0.05, n. s = not significant. **(H–K)** HUVECs were cultured either in MAN (5.5 mM) and HG + PA (33 mM HG+200 μM PA) medium in the presence or absence of Cel (100 nM) or Compound C (10 μM) for 48 h. The level of relative protein was evaluated by Western blot. The quantitative analysis of immunoblots **(I–K)**. Data shown in the graphs represent the means ± SD of independent experiments. #*p* < 0.05, ##*p* < 0.01 vs. HUVECs expose to MAN; *p < 0.01 vs. HUVECs expose to HG + PA, and *p* < 0.05 vs. HUVECs expose to Cel.

Consistent with this, we found that the low Nrf2 protein expression was restored (Figures 6A,C), and the expression of Keap1 remained unaffected by Cel and MG132 (a proteasome inhibitor) co-treatment (Figures 6A,D). Moreover, immunoprecipitation revealed that MG132 promotes the binding of Nrf2 to Keap1 in cells treated with HG + PA, indicating that Keap1 accumulates in cells and binds to Nrf2, causing it to undergo ubiquitination degradation in response to HG + *p*A. However, MG132 did not significantly increase the association of Nrf2 and Keap1 in Cel-treated cells, indicating that Cel affects Keap1 protein levels through the regulation of the non-proteasomal degradation pathway, ultimately inhibiting the binding of Keap1 to Nrf2, causing the latter to undergo ubiquitination degradation (Figures 6A,B).

**FIGURE 7:**
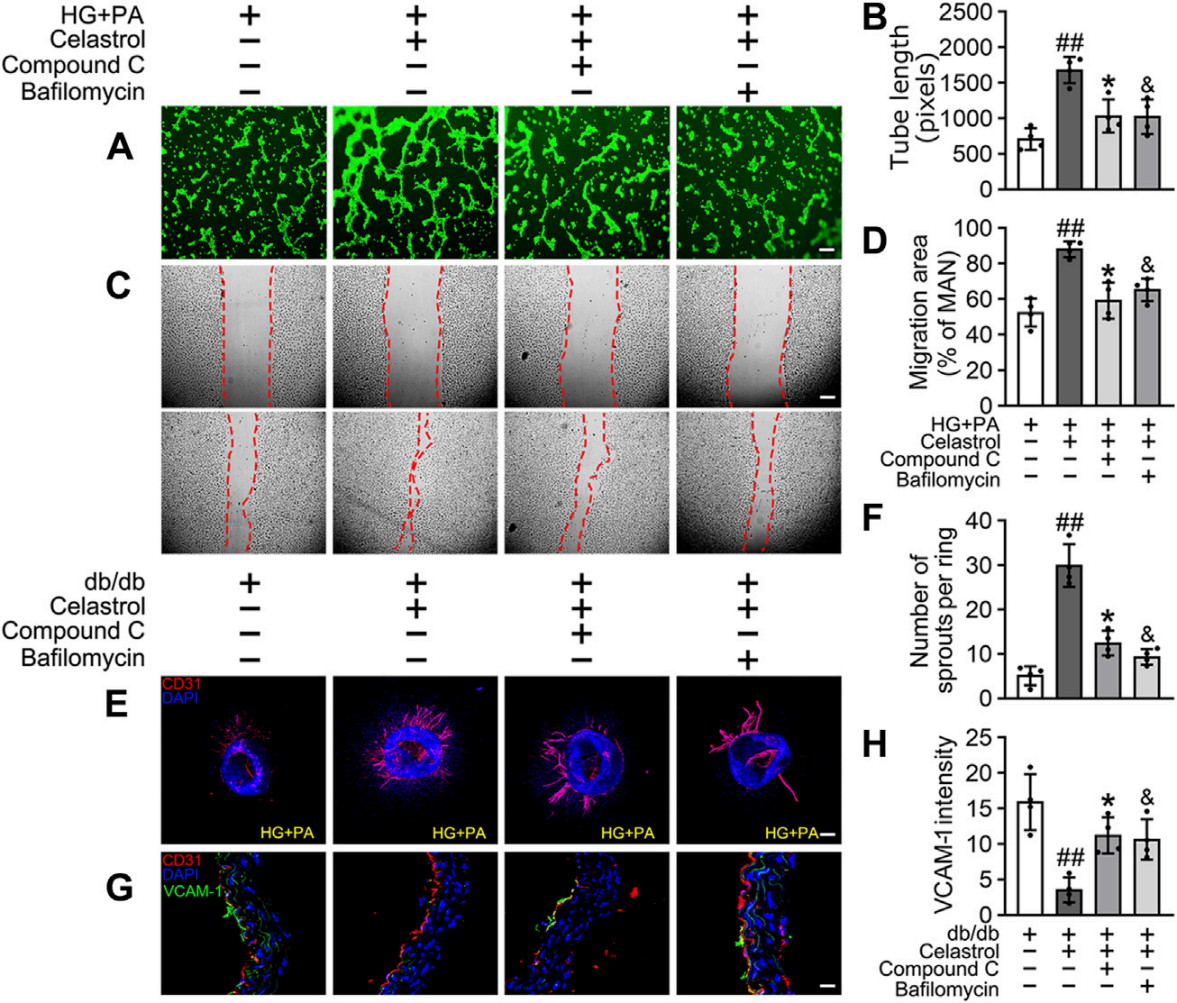
Cel ameliorates endothelial function via AMPK/autophagy pathway. **(A–D)** HUVECs were cultured in HG + PA (33 mM HG+200 μM PA) medium in the presence or absence of Cel (100 nM) or Compound C (10 μM) or Baf-A1 (20 nM) or MG132 (5 μM) for 48 h. **(A)** Capillary-like tube formation was assessed by matrigel angiogenesis assay in HUVECs. Scale bars = 85 μm. **(B)** Quantification of the tube length, and images of tube morphology were taken in four random microscopic fields per sample. **(C)** A scratch wound healing assay was performed in the presence of Mitomycin-C (10 μM). Cell monolayers were imaged at 0 and 36 h after wounding. Red vertical lines indicate the wound area borders. Scale bar = 65 µm. **(D)** Cell migration distances were measured based on the data. Data shown in the graphs represent the means ± SD of independent experiments. Data shown in the graphs represent the means ± SD of independent experiments. ##*p* < 0.01 vs. HUVECs expose to HG + PA; *p < 0.01, and *p* < 0.05 vs. HUVECs expose to HG + PA treatment with Cel. **(E–F)** Osmotic pumps containing Cel (100 μg/kg/day) was implanted intraperitoneally and were calibrated to release the drug for 28 days in db/db mice, and treatment with the absence or presence of Baf-A1 (10 mg/kg/2d) or Compound C (10 mg/kg/2d). **(E)** Representative images of aortic rings were pretreated with or without Baf A1 (20 nM) or Compound C (10 μM) for 2 h and then exposed to HG + PA in the presence or absence of Cel (100 nM). Scale bars = 350 μm. **(F)** Quantification of the number of sprouts. **(G)** Representative confocal images of inflammation stress marker VCAM-1 in aortal vascular endothelium. The red area (CD31) represented endothelium, the green area represented VCAM-1 positive staining and the nucleus was blue. Scale bars = 40 μm. **(H)** Quantification of the number of VCAM-1 staining. Data shown in the graphs represent the means ± SD of independent experiments. ##*p* < 0.01 vs. db/db mice; *p < 0.05, and *p* < 0.05 vs. db/db mice treatment with Cel.

Given that the accumulation of Nrf2 is accompanied by abundant autophagosomal degradation (Komatsu et al., 2010; Ichimura et al., 2013; Zhang et al., 2021; Taguchi et al., 2012), we next analyzed whether Cel increases Nrf2 expression by modulating autophagy. Co-treatment with Baf-A1, an inhibitor of autophagic degradation, blocked the effect of Cel on Nrf2 accumulation (Figures 6E–G). A previous study showed that Keap1 interacts with p62 (an autophagic adapter) and frees Nrf2 translocate to the nucleus (Taguchi et al., 2012). Consistent with this, in the present study, we found that the interaction between Keap1 and p62 in HUVECs was increased by HG + PA treatment and restored to normal by Cel administration (Figures 6E,F). Interestingly, the interaction between Keap1 and Nrf2 was increased by co-treatment with Baf-A1, even in the presence of Cel (Figures 6E,F), indicating that Baf-A1 inhibits the autophagy pathway involving Keap1 and p62, but does not reduce the expression of Keap1 protein, and increases the probability of Keap1 and Nrf2 binding, thereby increasing the ubiquitination and degradation of Nrf2. Thus, Nrf2 protein expression is reduced by Baf-A1 (Figures 6E,F). We also found that the effects of Cel on the phosphorylation of AMPK and autophagy were prevented by treatment with the specific pharmacological inhibitor of AMPK, compound C (Figures 6H–K). In addition, Cel had protective effects on tube formation (Figures 7A,B) and cell migration (Figures 7C,D), but these were abolished by compound C or Baf-A1 co-treatment.

To determine whether the protective effect of Cel against hyperglycemia-induced endothelial impairment is mediated via AMPK/p62-dependent autophagy in diabetic mice, compound C (10 mg/kg/2d, i. p.) or Baf-A1 (10 mg/kg/2d, i. p.) was administered. As expected, inhibition of the AMPK pathway or autophagy largely prevented the endothelial protective effect of Cel, as shown by a substantial increase in VCAM-1 expression in aortic vascular endothelium cells from db/db mice (Figures 7G,H) and an impairment in aortic ring sprouting (Figure 7E,F). These data suggest that Cel ameliorates the HG + PA-induced endothelial dysfunction by promoting the degradation of Keap1 in HUVECs, secondary to an effect on AMPK/p62-dependent autophagy, thereby liberating Nrf2 to translocate to the nucleus.

## Discussion

The 10th edition of the International Diabetes Federation Atlas estimated that there were 536.6 million people with diabetes in 2021 and that by 2045 there will be approximately 783.2 million adults with diabetes (Ogurtsova et al., 2022). The global prevalence of DFU has been reported to be 6.3%, higher in men than in women, and higher in patients with T2DM than in those with type 1 diabetes (6.4% vs. 5.5%, respectively) (Chen et al., 2023). Importantly, approximately 25% of patients with diabetes develop DFUs during their lifetime, and approximately 14%–24% of patients with DFUs ultimately require an amputation (Singh et al., 2005). In this study, the concomitant stimulation with high glucose (HG) and fatty acid (palmitic acid, PA) was used to mimic the *in vivo* T2DM-related hyperglycemia and hyperlipidemia condition on EC, as reported in different *in vitro* T2DM models (Alnahdi et al., 2019; Huang et al., 2021).

From a pathophysiological perspective, impaired wound healing in patients with diabetes is closely associated with inadequate angiogenesis (Okonkwo and DiPietro, 2017), which is caused by chronic inflammatory responses and impaired cellular responses to tissue hypoxia (Catrina and Zheng, 2016; Rehak et al., 2022). In addition, endothelial dysfunction, which includes endothelial cell dysfunction and a loss of endothelial cell barrier function, has also been reported in patients with DFUs (Zhang et al., 2022). Endothelial dysfunction is the most significant impairment affecting the microcirculation, and involves altered endothelial cell proliferation, thickening of the basement membrane, altered microvascular tone, and lower blood flow. Therefore, the effective treatment of chronic DFU necessitates improvements in endothelial function, angiogenesis, and immune function, and a reduction in inflammation, to promote tissue regeneration.

Cel is a quinone methide triterpenoid that is isolated from the traditional Chinese medicinal plant *Tripterygium wilfordii Hook f*. In recent years, Cel has received increasing attention for its potential therapeutic effects on inflammatory and metabolic disorders (Ma et al., 2015; Bian et al., 2016). Previous studies have shown that Cel reduces the obesity of HFD-fed diabetic obese (*db/db*) and leptin-deficient (*ob/ob*) mice by inhibiting endoplasmic reticulum stress and increasing STAT3-dependent leptin signaling (Liu et al., 2015). Furthermore, Cel reduces oxidative stress-related damage by increasing the activation of the Nrf2/antioxidase pathway (Divya et al., 2016). In addition, Cel has been shown to reduce the damage to vascular endothelial cells by reducing ROS production and the expression of pro-inflammatory molecules (Li et al., 2017). Therefore, Cel represents a potential treatment for DFUs. However, the detailed mechanism whereby Cel protects against DFU-associated endothelial cell dysfunction through the activation of Nrf2-associated extrinsic antioxidant defense mechanisms remains unclear.

Over the past few years, autophagy and oxidative stress have been shown to be intimately linked through complex signaling pathways. In the present study, we found that p62 activates the antioxidant transcription factor Nrf2 through a non-canonical pathway. The mechanism of this is completely independent of redox status and involves the recruitment of Keap1, an adapter protein of the cul3-ubiquitin E3 ligase complex, which is responsible for the degradation of Nrf2 (Komatsu et al., 2010; Ichimura et al., 2013). Consistent with this model, p62 binds to ubiquitinated protein aggregates and its affinity for Keap1 increases when it is phosphorylated at Ser351 (Ichimura et al., 2013). The induction of Keap1 autophagic degradation (Taguchi et al., 2012) liberates Nrf2 translocate in the nucleus. In the present study, we found that Cel ameliorates HG + PA-induced HUVECs injury by increasing activation of the autophagy-related p62-Keap1-Nrf2 signaling pathway, thereby increasing the activity of Nrf2, and reducing ROS production and the expression of pro-inflammatory cytokines. Additionally, Nrf2 has also been shown to regulate multiple aspects of key metabolic pathways in a tissue-specific manner, including lipid, carbohydrate, and amino acid metabolism, as well as iron transport and storage (Eid et al., 2019). Intriguingly and inconsistently, Nrf2^−/−^ mice have higher hepatic expression of Fgf21 than the wild type, resulting in improved glucose tolerance when fed a high-fat diet (Zhang et al., 2012; Slocum et al., 2016). In our study, endothelium-specific knockdown of Nrf2 had little effect on lipid and glucose metabolism in db/db mice treated with Cel. However, endothelium-specific Nrf2 deficiency abrogated the effect of Cel to ameliorate endothelial dysfunction and angiogenesis. Thus, we elucidate that Nrf2 in endothelial cells is a master regulator of vascular endothelial function improved by Cel.

Previous studies have shown that high glucose concentrations inhibit the activity of AMPK, which in turn stabilizes BCL2-BECN1, thereby inhibiting autophagy in cardiomyocytes (He et al., 2013). Interestingly, the atherosclerotic lesions of HFD-fed *Apoe* ^−/−^ mice are significantly worse than those of control mice because of impairment of endothelial autophagy, which implies that endothelial autophagy limits lipid accumulation in vessel walls (Torisu et al., 2016). However, several contradictory study found that cardiac-specific autophagy deficiency turned on Nrf2-mediated myocardial damage in the pressure-overloaded heart (Qin et al., 1979) and cardiac autophagy inhibition was critical for driving Nrf2-mediated ferroptosis in type 1 diabetic cardiomyocytes (Zang et al., 2020). Overall, this is most likely attributed to the fact that acute/controlled Nrf2 activation is protective, whereas chronic activation, which occurs in an autophagy-deficient setting, is detrimental, which have documented extensively elsewhere (Dodson and Zhang, 2017; Dodson et al., 2022). In the present study, we found that Cel increases the phosphorylation of AMPK and activates the autophagy-related p62-Keap1-Nrf2 pathway in the presence of high glucose and lipid concentrations, thereby ameliorating the oxidative stress and inflammation, and improving tube formation by endothelial cells and budding from aortic rings. Therefore, we hypothesized that if the autophagic flow could be restored in time, the activated Nrf2 would still play a role in anti-oxidative stress and cell protection.

There were several limitations to our study. Firstly, although HUVECs was the most widely used cells for the study of vascular function and repair (Guixé-Muntet et al., 2017; Cherubini et al., 2023), it is worth exploring the function of the additional dermal microvascular endothelial cells *in vitro* studies of diabetic wounds, which may behave slightly differently from HUVECs under high glucose or lipotoxicity. Secondly, wound contraction usually seen in rodent wound healing but minimal/absent in human wound healing. Thus, it is better to use appropriate methods to prevent wound contraction (staying wet or using semi-occlusive wound dressing). Although we could confidently demonstrate the protective effect of Cel on diabetic wound healing through re-epithelialization, the contractive effect of skin wound healing may have increased the experimental uncertainty to some extent, even if the environment of each group in this study remained the same. Thirdly, although Cel could promote angiogenesis associated with anti-oxidative stress and anti-inflammation in T2D mice, it could not eliminate that completely. These results suggest that Cel has partial effect on improving wound healing following diabetic foot ulcers. It might be worth exploring whether Cel has better vascular protection on other complications of diabetes.

**FIGURE 8:**
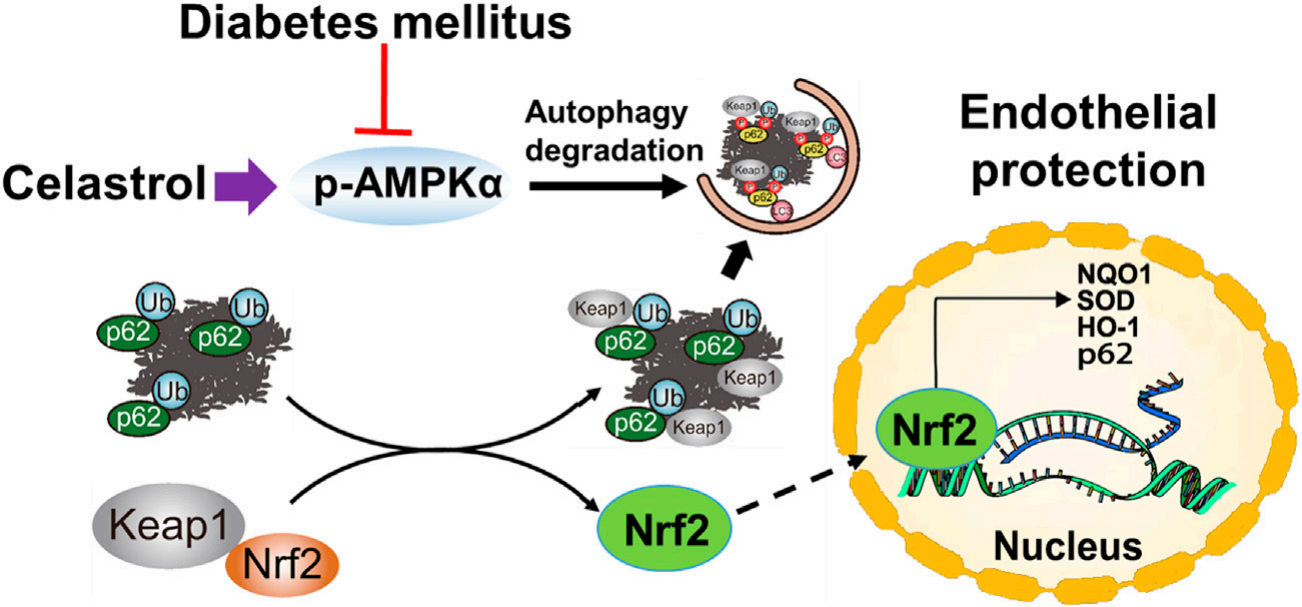
Schematic illustration of the protective effects of Cel on HUVECs under HG + PA conditions. HG + PA decreases the expression of Nrf2 in HUVECs and induces oxidative stress, which impairs the survival and angiogenic function of HUVECs. Under HG + PA conditions co-treatment with Cel improves HUVECs survival and function predominantly by Nrf2 activation mediated by increasing phosphorylation of AMPK and autophagic degradation of p62/Keap1.

In summary, in the present study, we have shown for the first time that low-dose Cel does not affect the ability of cells to form tubes or angiogenesis under normal conditions. However, the protective effects of Cel against hyperglycemia and hyperlipidemia-induced endothelial injury may be at least in part the results of a maintenance of normal oxidative status and the consequent inhibition of inflammation. In addition, Cel was shown to reduce hyperglycemia/hyperlipidemia-induced inflammation through the AMPK-dependent p62-Keap1-Nrf2 signaling pathway (Figure 8). Although the poor water solubility, short plasma half-life, and high systemic toxicity of Cel greatly hinder its clinical use (Geng et al., 2022), these findings may suggest a new therapeutic approach for the short-term, targeted treatment of refractory diabetes-related skin ulcers and vascular defects in the future. Importantly, there have been many studies on the delivery of Cel by nanomaterials to ameliorate inflammation (An et al., 2020; Wu et al., 2022), we believe that the novel nanocomposites could overcome the poor bioavailability and short half-life of Cel and could be a good candidate for topical application in diabetic foot ulcers.

## Data Availability

The original contributions presented in the study are included in the article/Supplementary Material, further inquiries can be directed to the corresponding authors.
