# Supplementary material for: Celastrol alleviates diabetic vascular injury via Keap1/Nrf2-mediated anti-inflammation
Source: Front Pharmacol. 2024 May 31;15:1360177. doi: 10.3389/fphar.2024.1360177 (PMC11176472; doi:10.3389/fphar.2024.1360177)

**Celastrol alleviates diabetic vascular injury via Keap1/Nrf2-mediated anti-inflammation**

**Supplemental Table 1. The blood fasting glucose of diabetic mice (mM)**

|  | db/dm (n=4/group) | db/db  (n=4/group) | | | | |
| --- | --- | --- | --- | --- | --- | --- |
|  |  | Vehicle | Celastrol | | | |
|  |  |  | - | AAV9-sh-Nrf2 | Compound C | Baf-A1 |
| 7-week old | 4.5±0.6 | 13.1±2.9 | 12.4±2.3 | 13.7±1.4 | 14.7±1.9 | 12.8±2.1 |

**Supplemental Table 2.** qRT-PCR primer sequences

| *SOD2* | GTCACCGAGGAGAAGTACCAGGAGGCG |
| --- | --- |
|  | CGCCTCCTGGTACTTCTCCTCGGTGAC |
| *HO1* | GCCCCAGGATTTGTCAGAGG |
|  | GGAGGCCATCACCAGCTTGAA |
| *CAT* | CCTTCTTGTTCAGGATGTGGTTTTC |
|  | CATGTGTGACCTCAAAGTAGCCAAA |
| *NQO1* | GAAGAGCACTGATCGTACTGGC |
|  | GGATACTGAAAGTTCGCAGGG |
| *NQO2* | GTACTCATTGTCTATGCACACCA |
|  | TGCCTGCTCAGTTCATCTACA |
| *TNF-α* | CATCTTCTCAAAACTCGAGTGACAA |
|  | TGGGAGTAGATAAGGTACAGCCC |
| *IL-8* | GCCAACACAGAAATTATTGTAAAGCTT |
|  | AATTCTCAGCCCTCTTCAAAAACTT |
| *IL-6* | ACTCACCTCTTCAGAACGAATTG |
|  | CCATCTTTGGAAGGTTCAGGTTG |
| *IL-1β* | TGGGAGTAGATAAGGTACAGCCC |
|  | GTAGTGGTGGTCGGAGATTCG |
| *Actb* | GCACAGAGCCTCGCCTT |
|  | GTTGTCGACGACGAGCG |

**Supplementary Figures**


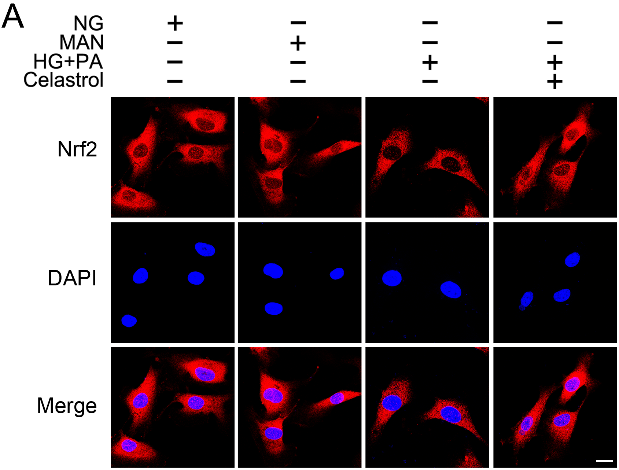


**Supplementary Fig 1.** **Cel promoted Nrf2 accumulation in the nucleus.** (A) The nuclear localization of Nrf2 was measured by immunofluorescent staining of HUVEC, with red areas representting Nrf2 and nuclei in blue. Scale bars=20 μm.


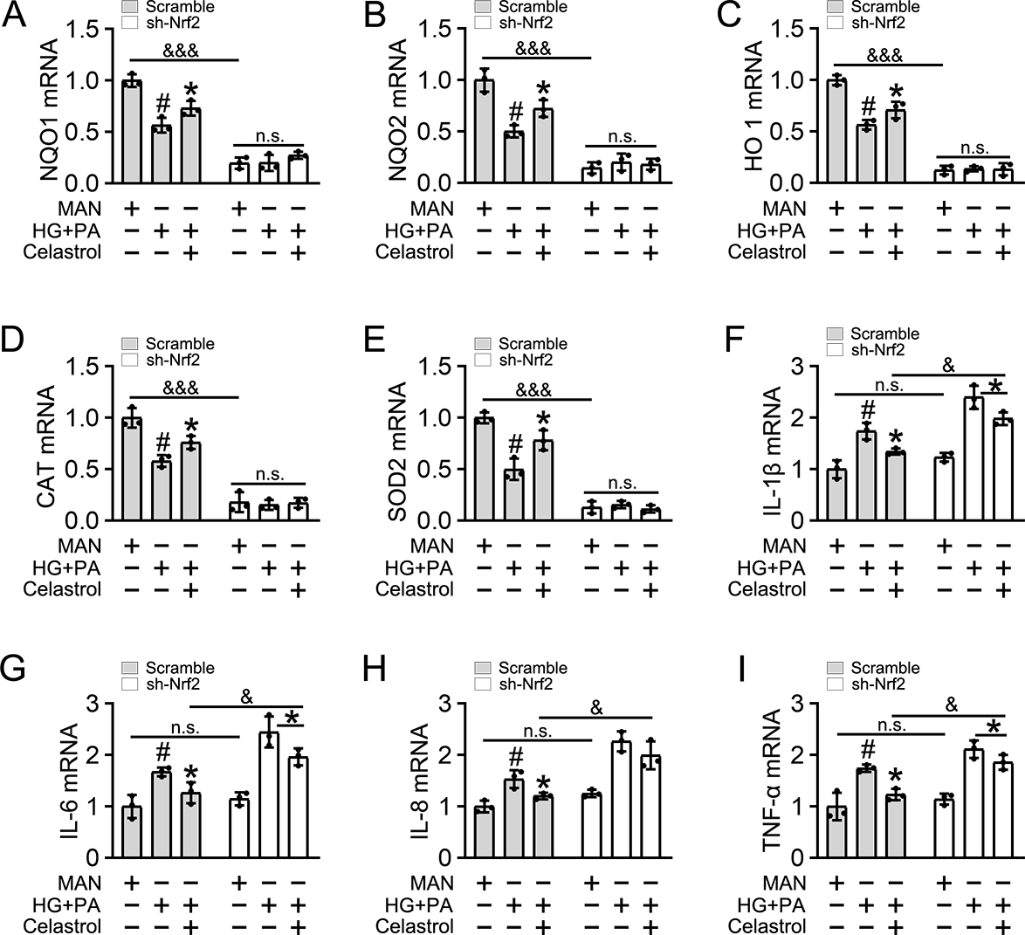


**Supplementary Fig 2. Nrf2 sh-RNA abolished the anti-inflammatory effect of Cel.** HUVECs were transfected with Nrf2 shRNA (sh-Nrf2) or scramble shRNA (scramble) in the presence or absence of Cel for 48 h. (A-E) Nrf2 downstream target genes were quantified using qRT-PCR. (F-I) NF-κB downstream target genes were quantified using qRT-PCR. Data shown in the graphs represent the means ± SD of independent experiments. n.s. = not significant, #p < 0.05 vs. HUVECs expose to MAN,*p < 0.05 vs. HUVECs expose to HG+PA, &p < 0.05, &&&p < 0.001.


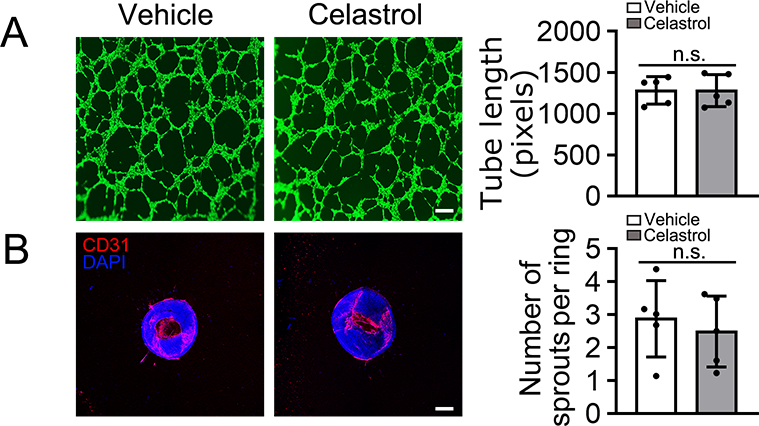


**Supplementary Fig 3.** **Cel had a very small effect to enhance tube formation and aortic ring sprout under basal conditions.** (A) Evaluation of tube formation in HUVECs by matrigel angiogenesis assay. Scale bars=300 μm. Quantification of the tube length (left), n.s. = not significant. (B) db/dm mice were surgically isolated, cleaned, and sectioned to form 0.5 mm rings. Next the rings were embedded and cultured with or without Cel. All aortic rings cultured without VEGF. Representative images and quantification of aortic rings sprouting (left). n.s. = not significant.


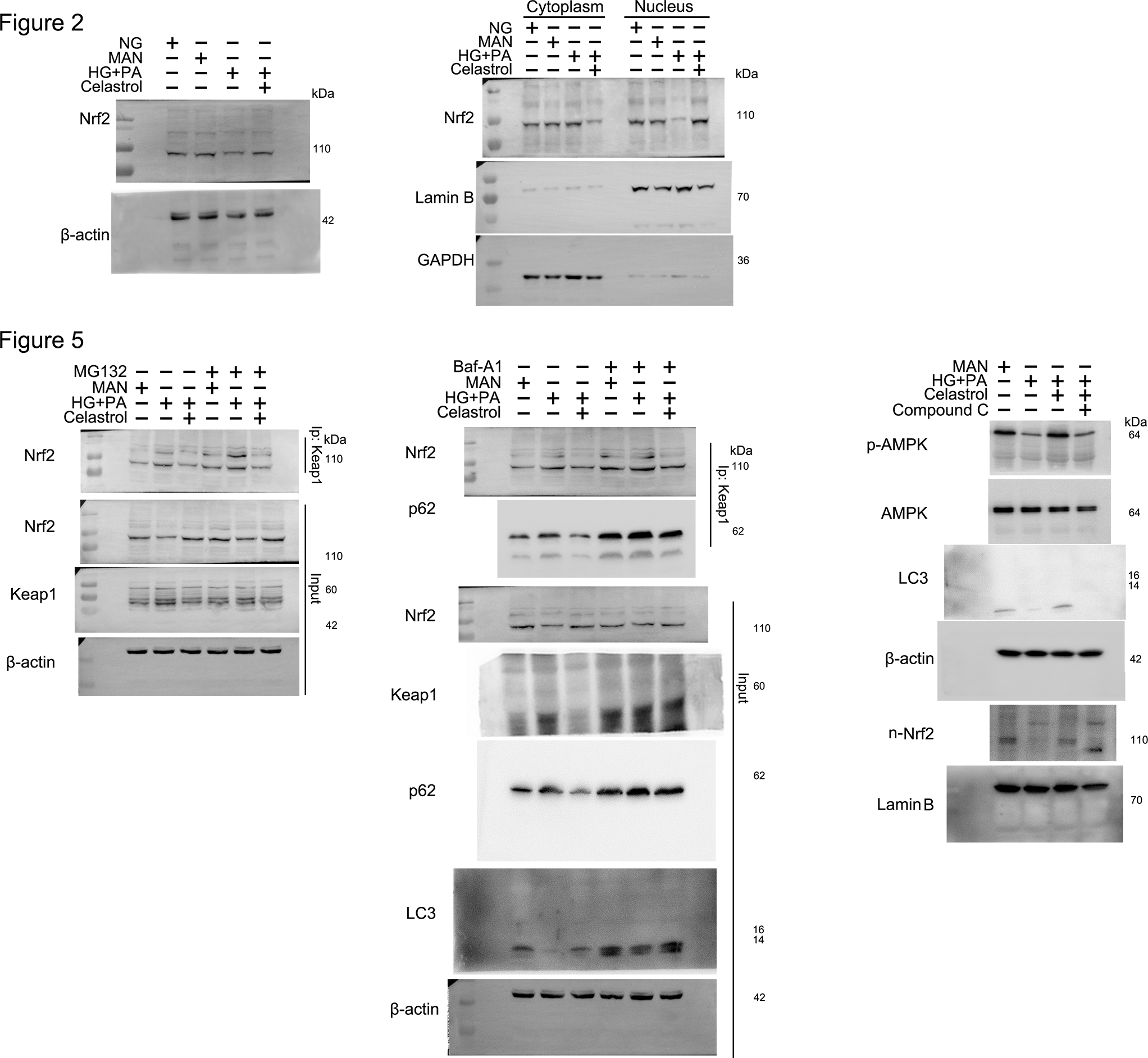

Supplement: Supplementary file 2 [file DataSheet1.docx]
